# Innovative Sericin-Based Film-Forming Gel for Wound Healing: Development and Performance Evaluation

**DOI:** 10.3390/polym17091246

**Published:** 2025-05-03

**Authors:** Suprawee Wongtechanon, Chayanee Noosak, Pavarish Jantorn, Papitchaya Watcharanurak, Piyawut Swangphon, Warapond Wanna, Dennapa Saeloh Sotthibandhu

**Affiliations:** 1 Faculty of Medical Technology, Prince of Songkla University, Songkhla 90110, Thailand; fongsuprawee@gmail.com (S.W.); chayanee.no@wu.ac.th (C.N.); pavarish.j@psu.ac.th (P.J.); papitchaya.w@psu.ac.th (P.W.); piyawut.s@psu.ac.th (P.S.); 2 Division of Biological Science, Faculty of Science, Prince of Songkla University, Songkhla 90110, Thailand; waraporn.wa@psu.ac.th; 3 Department of Medical Technology, School of Allied Health Sciences, Walailak University, Nakhon Si Thammarat 80160, Thailand

**Keywords:** film-forming gel, polyvinyl alcohol, sericin, wound healing

## Abstract

The development of effective wound dressings remains a critical challenge in medical treatments, requiring materials that promote healing, minimize infection, and enhance tissue regeneration. This study evaluated the wound-healing potential of sericin-based film-forming gels. Six formulations were developed by combining varying concentrations of sericin, a protein derived from silk cocoons, with polyvinyl alcohol (PVA). These formulations were evaluated for physical properties including drying time, pH, spreadability, stability, swelling ratio, flexibility, and adhesion. Film-forming gel is an attractive option for wound dressing due to its flexibility, adhesion, and infrequent reapplication. The F4 formulation (1% sericin) demonstrated superior performances in drying time, spreadability, stability, swelling ratio, flexibility, and skin adhesion, was easy to apply, and formed a stable film on drying. Biological evaluations showed that F4 exhibited excellent compatibility with skin fibroblast cells, maintained a suitable pH, and significantly promoted cell proliferation and migration. The F4 formulation also demonstrated anti-inflammatory effects by inhibiting iNOS expression and nitric oxide production, offering mechanical stability, biological activity, and ease of use with significant potential for treating acute and chronic wounds.

## 1. Introduction

Wounds are physical injuries that result in the breaking of cells and membranes [1]. The disruption of structure and function in healthy skin creates a cavity that requires repair. The economic and social burden of wounds is significant. Medicare costs associated with acute and chronic wounds were estimated at $28.1–$96.8 billion in 2018 [2]. In the United States, medical expenses for surgical scars and injuries were around $12 billion, while burn wounds amounted to $7.5 billion each year [3]. The World Health Organization reported that over 300,000 people die from skin injuries annually [4]. Wound healing is a biological progression that involves four phases: homeostasis, inflammation, proliferation, and remodeling [5]. Skin tissue possesses the ability to self-repair, but certain wound types, including diabetic ulcers, burn injuries, and large surface areas or deep wounds, exhibit impaired healing processes [6]. A multifunctional wound dressing is crucial for supporting the wound-healing process. The ideal wound dressing should be non-allergenic and minimize side effects with the ability to absorb excess wound exudate, maintain skin moisture, and stimulate wound-healing mechanisms [5].

Natural extracts have recently attracted interest in tissue engineering because of their biodegradability, biocompatibility, and bioresorbability [7]. Several natural products have shown potential as effective bioactive materials for wound healing and anti-inflammatory applications, such as gotu kola, young coconut, and silk cocoons [8,9,10]. Sericin extracted from silk cocoons has been studied for use in cosmetics and tissue engineering due to its excellent biological activities, biocompatibility, and low immunogenicity [11]. Sericin is usually discarded as waste from the textile industry, causing environmental contamination [4]. Previous studies indicated that sericin has remarkable properties as a wound dressing biomaterial, including anti-inflammatory effects, the promotion of cell proliferation, and the enhancement of collagen synthesis through activation of fibroblasts [12,13]. Sericin demonstrated cytoprotective and mitogenic effects on fibroblasts and keratinocytes, highlighting its potential as an option for the formulation of advanced skin and tissue regenerative materials [4]. However, sericin has poor mechanical strength, which could be improved by the combination with other biomaterials. Sericin contains hydroxyl, carbonyl, and amino groups in its side chains, allowing easy cross-linking, blending, and copolymerization with various materials [4,14]. Polyvinyl alcohol (PVA) is a synthetic polymer known for its biocompatibility, biodegradability, and affordability. PVA has outstanding mechanical and gas barrier properties, chemical resistance, and good film-forming ability [15,16]. Thus, the combination of sericin and PVA could improve the mechanical performance of sericin-based biomaterials used in wound dressing.

Various forms of sericin/PVA biomaterials have been developed for innovative wound-dressing applications, such as hydrogels, nanofibers, and films [6,15,17]. Sericin films exhibited a slow degradation rate, with increased cell adhesion and viability of the fibroblasts [10]. However, the film form has low stability, which impacts applications, depending on the specific types of wounds or individual patients. A liquid film-forming gel that transformed into a stable solid film covering the skin on drying would prolong the effectiveness of the active ingredients. A film-forming gel is also easy to apply and provides good flexibility and adhesion to the skin surface with infrequent reapplication [18].

This study developed sericin/PVA-based film-forming gels for wound-healing applications. The physical performance of formulations with various ratios of sericin were characterized and evaluated for wound dressing, including drying times, pH, spreadability, stability, and swelling ratio. The biological effects of the films were assessed by investigating their cytocompatibility with fibroblast cells, wound-healing activities, and anti-inflammatory properties.

## 2. Materials and Methods

### 2.1. Preparation of Sericin Extract

Sericin was extracted following the method used in a previous study [19]. In brief, 32 g of dry silkworm cocoons were boiled in 1 L of distilled water at 100 °C for 90 min. The sericin solution was freeze-dried using a Coolsafe Touch freeze dryer (Labogene, Bjarkesvej, Denmark).

### 2.2. Preparation of Film-Forming Gel Formulations

Polyvinyl alcohol (10% *w*/*v*) (PVA; Loba, Maharashtra, India) was dissolved in distilled water at 95 °C. Subsequently, 0.25%, 0.5%, and 1% (*w*/*v*) of sericin solution were added to the PVA solution and stirred continuously. Glycerol and 95% ethanol were immediately added to the sericin/PVA solution and mixed homogeneously. The concentrations of components in each sericin-based film-forming gel formulation are shown in Table 1.

### 2.3. Determination of Film Evaporation Times on the Skin and the Slide

The drying times of the films were determined on the skin and the slide. One hundred microliters of each formulation were applied to either the skin of a stillborn piglet or a glass slide, each with an area of 2.5 × 2.5 cm^2^. Drying times were recorded by gently touching the skin or glass slide, and the integrity of the dried film on the skin was subsequently assessed [20].

### 2.4. Determination of pH

The pH values of all the formulations were measured in triplicate with a Eutech^TM^ pH 700 meter (Thermo Scientific, Waltham, MA, USA).

### 2.5. Determination of Spreadability

One gram of each formulation was dropped onto a glass plate and covered with another glass plate. The time taken to spread was recorded, with the spreadability calculated using the following equation:

(1) $$Spreadability=\frac{W\times A}{T}$$

where W is the weight of the formulation, A is the area of the glass plate, and T is the spread time.

### 2.6. Assessment of Stability

All formulations were kept at 4 and 25 °C for 30 and 60 days. The evaporation time on the skin and the glass slide, pH, and spreadability were recorded and analyzed.

### 2.7. Evaluation of Swelling Ratio

A 50 μL aliquot of all the formulations was dropped onto a glass slide with an area of 1 × 1 cm. The formulations were dried in an incubator at 60 °C overnight. The films were then weighed. The dry weight was recorded before the films were immersed in phosphate-buffered saline (PBS) and incubated at 37 °C. The films were weighed at 1, 2, 4, 8, and 12 h. The swelling ratio was calculated using the following equation:

(2) $$Swelling\;ratio\left(\%\right)=(\frac{Ws-Wd}{Wd})$$

where W_s_ is the weight of the swelled sample and W_d_ is the weight of the dry sample.

### 2.8. Collection of Film-Forming Gel Supernatant

The F4, F5, and F6 formulations were dried in a 24-well plate. Dulbecco’s Modified Eagle Medium (DMEM; Gibco, Grand Island, NY, USA) containing 10% fetal bovine serum (FBS; Gibco, Grand Island, NY, USA) and 1% antibiotic–antimycotic (Gibco, Grand Island, NY, USA) was added to the film surface and incubated under 5% CO_2_ at 37 °C. At specific times, the film supernatant was collected for the following experiments.

The supernatants of the film-forming gel collected at 3, 6, 12, 18, and 24 h were measured at a wavelength of 420 nm to determine the sericin release.

### 2.9. Fibroblast Cell and Macrophage Cell Culture

Fibroblast L929 cells and macrophage RAW 264.7 cells were cultured in DMEM medium containing 10% FBS and 1% antibiotic–antimycotic under 5% CO_2_ at 37 °C. After reaching confluence, the cells were harvested using Trypsin-EDTA (Gibco, USA).

### 2.10. Evaluation of Cell Viability

The cell viability was determined by the MTT assay. Fibroblast L929 cells were seeded at a density of 2 × 10^4^ cells/well into a 96-well plate and incubated overnight under the condition previously described. The cells were treated with the film-forming gel supernatants collected at 6, 12, and 24 h. After 24 h of incubation, the cell viability was analyzed using MTT reagent (Alfa Aesar, Heysham, Lancashire, UK), and the absorbance was measured at a wavelength of 570 nm. The total cell viability was calculated by the following equation:

(3) $$Cell\;viability\left(\%\right)=\frac{AT}{AC}\times 100$$

where AT is the absorbance of treated cells, and AC is the absorbance of untreated cells.

### 2.11. Scratch Wound Assay

Fibroblast L929 cells were seeded at a density of 1 × 10^5^ cells/well in 24-well plates and cultured for 24 h under 5% CO_2_ at 37 °C. The cell monolayer was scratched with a sterile tip across the center of each well. The cells were washed with the culture medium to remove the detached cells and subsequently treated with the film-forming gel supernatant collected at 24 h. The cells with the culture medium were used as a control. Wound-healing efficiency was monitored at 0, 12, and 24 h. The scratch closure rate was calculated using the following equation:

(4) $$Scratch\;closure\;rate=\frac{A_{t0}-A_{t}}{A_{t0}}\times 100$$

where A_t0_ is the scratch area at time 0 h, and A_t_ is the comparable scratch area at 12 and 24 h.

### 2.12. Determination of Nitric Oxide Concentration

Nitric oxide (NO) production was determined according to the nitrite levels by the modified Griess assay. The macrophage RAW 264.7 cells were cultured on high glucose-DMEM (Sigma Aldrich, St. Louis, MO, USA) supplemented with 10% FBS and 1% antibiotic–antimycotic at 37 °C under 5% CO_2_. The cells were seeded at a density of 5 × 10^4^ cells/well into a 96-well plate. After incubation, the cells were pre-treated with the film supernatant collected at 6, 12, and 24 h for 4 h. Lipopolysaccharide (LPS) at 1 μg/mL was added to each well and further incubated for 24 h. Subsequently, the culture medium from each well was collected and incubated with a modified Griess reagent (Sigma-Aldrich, USA) for 15 min in the dark. The absorbance was measured at a wavelength of 540 nm to calculate the NO concentration.

### 2.13. Real-Time Quantitative PCR Analysis

The macrophage RAW 264.7 cells were seeded in a 96-well plate at a density of 5 × 10^4^/well and cultured for 24 h under 5% CO_2_ at 37 °C. The cells were pre-treated with the F4 supernatant collected at 12 h for 4 h. Then, LPS was added to each well and further incubated for 24 h. Cells treated with dexamethasone (10 μM) containing LPS (1 μg/mL) were used as a control. The total RNA concentration was extracted using a total RNA mini kit (Geneaid Biotech Ltd., New Taipei City, Taiwan), and the purified RNA samples were detected by a Nanodrop spectrophotometer (Thermo Scientific, USA). The total RNA of each sample was used for reverse transcription into cDNA using a RevertAid First Strand cDNA synthesis kit (Thermo Scientific, USA). Quantitative reverse transcription PCR (RT-qPCR) was determined using a Bio-Rad real-time PCR CFX96 (Bio-Rad, Hercules, CA, USA). The RT-qPCR analysis was performed using an EvaGreen PCR reagent kit (Solis Biodyne, Tartu, Estonia). The relative amount of gene expression was determined using the 2^−△△Cq^ method. The specific primers used in this study are shown in Table 2.

### 2.14. Western Blot Assay

The RAW 264.7 cells were seeded in 6-well plates at a density of 5 × 10^5^ cells/well. The cells were pre-treated with the F4 supernatant collected at 12 h for 4 h and stimulated with LPS (1 μg/mL) for 24 h. The total protein in the cells was extracted using a radioimmunoprecipitation assay buffer (RIPA; Servicebio, Wuhan, Hubei, China), with protein concentrations determined using a Pierce BCA protein assay kit (Thermo Scientific, Rockford, IL, USA). Equal quantities (20 μg) of protein samples were separated by SDS-PAGE and transferred into a polyvinylidene difluoride (PVDF) membrane (Servicebio, Wuhan, Hubei, China). The membranes were probed with primary antibodies including ant-iNOS and anti-β-actin (Cell Signaling, Danvers, MA, USA) overnight, and the horseradish peroxidase-linked secondary antibody (Cell Signaling, Danvers, MA, USA) for 1 h at room temperature. β-actin was used as an internal control. Protein bands were detected by enhanced chemiluminescence (ECL) Western blotting detection reagent (Servicebio, Wuhan, Hubei, China) and visualized using ChemiDoc Imaging (Bio-Rad, Hercules, CA, USA).

### 2.15. Statistical Analysis

The values shown are the means of the three wells from three independent experiments. Statistical significance was determined using a two-way Student’s *t*-test for independent means using Microsoft Excel. Data are presented as the mean ± SD of three independent experiments, with *p* < 0.05 considered statistically significant.

## 3. Results and Discussion

### 3.1. Characteristics and Physical Properties of the Film-Forming Gels

#### 3.1.1. Characteristics of the Film-Forming Gel

Sericin and PVA film-forming gels were developed for wound-healing applications. The combination of sericin with PVA was chosen due to its favorable properties for wound healing. While other combinations of sericin with different polymers exist, PVA was selected for its unique advantages. For example, although chitosan contains NH_2_ groups that offer antimicrobial benefits, it tends to be less flexible and has lower film-forming ability than PVA [22]. Alginate, which contains COOH groups and is highly absorbent, does not provide the same stability or film-forming strength as PVA [23]. Gelatin, with its amino acid residues, supports cell attachment but may be less stable and degrade more easily in moist conditions [24]. In contrast, PVA, which contains hydroxyl (-OH) groups, offers excellent film-forming ability, flexibility, and moisture retention, all of which are crucial for maintaining an optimal environment for wound healing [25]. Our previous study demonstrated that the combination of PVA and sericin significantly improved the mechanical and biological properties of hydrogels, enhancing their effectiveness in supporting tissue repair in orthopedic applications [26]. When combined with sericin, which contributes bioactive properties that promote tissue regeneration, PVA provides a balanced combination of structural integrity and biological activity, making it an ideal choice for wound-healing applications.

Sericin was incorporated as a bioactive component due to its proven biological activities, including stimulation of collagen synthesis and promotion of fibroblast migration [27]. Additionally, glycerol was added to the formulation as a plasticizer to improve the flexibility of the dried film and prevent cracking over time [28]. It also helped in moisture retention and influenced phase separation between sericin and PVA [29]. Furthermore, ethanol was included to enhance permeability and reduce the drying time of the gel, facilitating faster film formation upon application.

**Figure 1:**
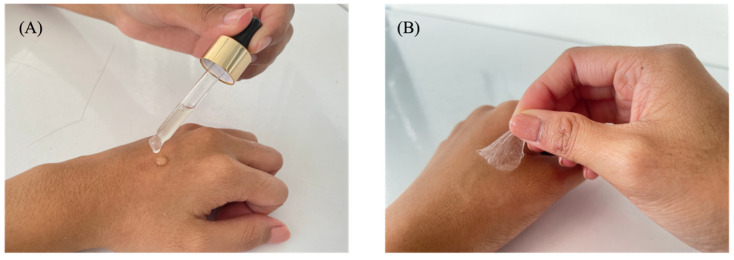
Representative images of sericin-based film-forming gel. (**A**) Gel stage, (**B**) Film stage.

All dry films obtained from the film-forming gel solution exhibited transparency with a light-yellow color and formed a complete film with no cracking or flaking (Figure 1). The characteristics of the different sericin-based film-forming gel formulations are shown in Table 3. The film-forming gel formulations were applied to the skin and slide to determine the drying time. The evaporation test performed on the slide negated various skin factors that influenced drying time, such as skin humidity and individual skin characteristics, providing a more standardized and reproducible measurement of drying time.

The formulations F4, F5, and F6 exhibited shorter drying times compared to F1, F2, and F3 on the skin (3–4 min vs. 4–5 min) and on the slide (6–7 min vs. 8–10 min), Table 3. This difference was attributed to the higher ethanol content in the F4, F5, and F6 formulations, which accelerated the evaporation process. Similar trends were observed in other studies. For example, in an etoricoxib film-forming gel study, drying times ranged from 3–6 min, with 10 out of 17 formulations drying at similar rates and comparable to our F1–F6 formulations [30]. In a terbinafine hydrochloride-loaded film-forming gel study, the first two formulations dried faster (2–3 min), while the remaining formulations had similar drying times [31]. These results mirrored our findings, where formulations with higher ethanol content (F4, F5, F6) dried faster than those with lower ethanol content (F1, F2, F3), suggesting that the ethanol content played a significant role in reducing drying times across different formulations and supporting the idea that a higher ethanol volume promoted faster evaporation [32]. By contrast, a study on a film-forming polymeric dispersion containing *Centella asiatica* reported a longer drying time of 10–15 min to form a film [33], slower than the drying times observed in our study. This result highlighted how different formulation compositions can significantly influence the drying behavior of film-forming gels.

The pH of the formulations played an important role in their skin compatibility. Topical formulations should be acidified to achieve a pH level ranging between 4.1 and 5.8 [34]. As shown in Table 3, the pH values of the F1, F2, and F3 formulations were lower than those of the F4, F5, and F6 formulations, which ranged from 5.2 to 5.4. The pH of sericin was recorded as 5.97 ± 0.04, with PVA 5.12 ± 0.03. The pH values of glycerol and ethanol were 3.35 ± 0.02 and 6.76 ± 0.02, respectively. The F4, F5, and F6 formulations, which contained high volumes of ethanol, demonstrated increased pH values. A previous study recorded the natural pH of the skin of 330 human volunteers as 4.0 to 7.0 [35]. Therefore, the obtained skin pH could be applied in the development of effective topical products. All the formulations revealed suitable pH values, suggesting compatibility with the skin while minimizing the risk of irritation.

Spreadability is a characteristic used to assess the spread of film-forming gel on the skin surface. A consistent standard dose of a medicated formulation must be delivered to the skin to ensure the efficacy of a topical treatment. The spreadability values of the F1 and F4 formulations were lower than those of the other four formulations because the sericin extract became a gel and formed a film [36]. Generally, films with low spreadability tend to be more effective because they are easy to apply and enhance adherence to the skin during use [37]. Films with low spreadability reduce the risk of unintended movement from the applied area, which is an advantage for targeted treatments. The results demonstrated that the F1 and F4 formulations exhibited low spreadability, indicating ease of application and enhanced usability on the skin.

**Figure 2:**
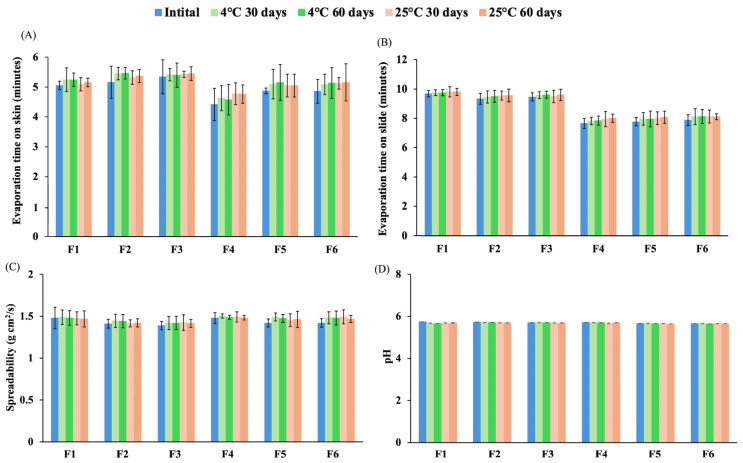
Physical stability of sericin-based film-forming gel under different conditions. (**A**) Evaporation time on the skin, (**B**) Evaporation time on the glass slide, (**C**) Spreadability, and (**D**) pH of the formulations.

The stability of the formulations was compared at three time points: initial time, 30 days, and 60 days, under controlled temperature conditions of 4 °C and 25 °C. All the formulations presented homogeneous gels. The dry films obtained from the film-forming gel solutions were light transparent yellow, with no cracking or flaking in the complete film. Figure 2 shows the properties of film-forming gels under various conditions. The drying time of the film on the skin ranged from 3.5–5 min, whereas the drying time on the glass slide was stable at 7–9 min, exhibiting longer drying time on the skin and the slide compared to the initial time (Figure 2A,B). The spreadability of the formulations remained consistent with the initial time at all temperatures after 30 days and 60 days of storage (Figure 2C). The pH of the formulations exhibited stability over 60 days, maintaining values within the range of 5–6 (Figure 2D). Ethanol plays a crucial role in enhancing the self-preserving system, contributing to the stability and safety of the final product [38]. A previous study demonstrated that ethanol could be used as a solvent, skin enhancer, and preservative of hydroalcoholic gels for topical administration [39]. Glycerol was also reported as a plasticizer and stabilizer solution for film formation and gel structure [40,41]. In the formulation, alcohol acted as a preservative, while glycerol functioned as a plasticizer and stabilizer, contributing to the overall stability and integrity of the product. The findings indicated that 4 °C and 25 °C were the optimal storage conditions for all the formulations to prevent degradation or loss of effectiveness over time.

#### 3.1.2. Swelling Capacity of the Film-Forming Gels

Wound dressing biomaterials should effectively absorb excess exudate from the wound while maintaining a moisture-proof environment to support the healing process [5]. Our formulations contain glycerol, which acts as a moisturizer by keeping the skin moist, protecting it from excessive drying, and preventing moisture loss through the formation of a protective barrier [42]. Sericin, also present in the formulations, is rich in polar amino acids, including serine, aspartic acid, and glutamic acid. These amino acids contribute to sericin’s hydrophilic nature, enhancing its ability to absorb and retain moisture [43,44].

**Figure 3:**
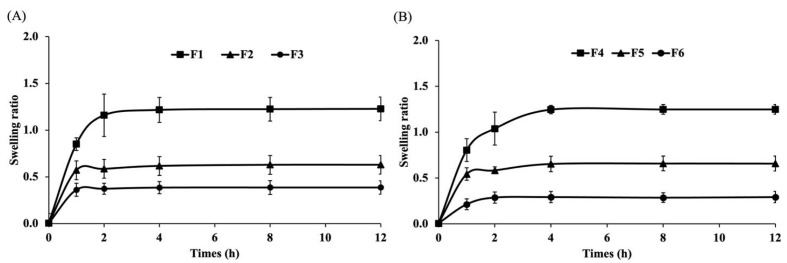
Swelling ratios of sericin-based film-forming gels at different times. (**A**) F1, F2, and F3 and (**B**) F4, F5, and F6.

To evaluate the water absorption capacity of each formulation, the swelling ratio was measured. As shown in Figure 3, the F1 formulation exhibited the greatest swelling ratio, followed by F2 and F3 (Figure 3A), while F4 demonstrated more swelling than F5 and F6 (Figure 3B). Formulations F1 and F4, which contained 1% sericin, showed higher swelling ratios compared to those with 0.5% and 0.25% sericin. These findings highlight the significant role of sericin in enhancing water absorption, which may contribute to the effective management of exudate in wound care. Consistent with previous studies, sericin enhanced the swelling capability of the hydrogel, showing an increase of up to 15% after immersion in PBS for 4 h [45].

### 3.2. Biological Activities of the Film-Forming Gels

#### 3.2.1. Effects on Wound Healing

**Figure 4:**
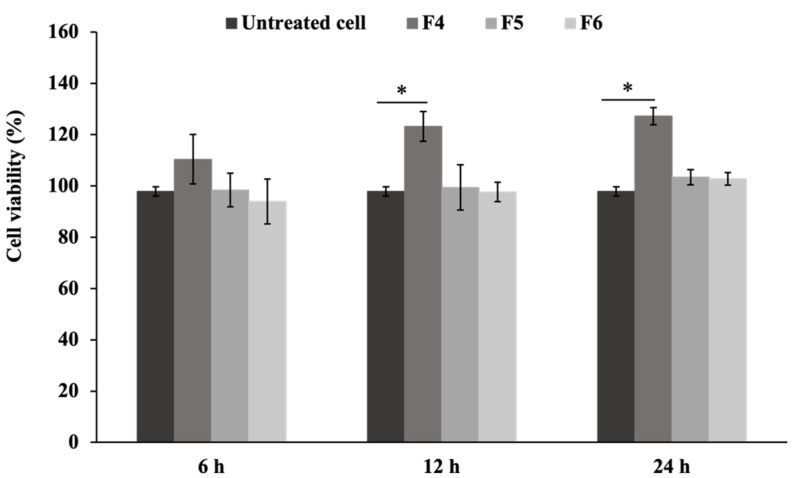
The effects of F4, F5, and F6 supernatants on the viability of the L929 cell line at 6, 12, and 24 h (* *p* < 0.05 compared to untreated cells).

Based on the gel characteristics and physical testing experiments, the F4, F5, and F6 formulations were selected for subsequent biological evaluation. The MTT assay was carried out to assess the cytotoxicity of film-forming gels on fibroblast cells. The supernatants from the F4, F5, and F6 formulations were used to treat L929 fibroblast cells, with the corresponding percentages of cell viability shown in Figure 4. The formulations revealed no cytotoxicity, with F4 significantly enhancing cell proliferation compared to the untreated cells after 12 and 24 h (*p* < 0.05). Sericin enhanced fibroblast cell proliferation without inducing cytotoxic effects, potentially improving wound healing and skin regeneration applications in biomedical fields [10]. The F4 formulation containing the highest concentration of 1% sericin extract showed enhanced cell proliferation compared to F5 and F6.

**Figure 5:**
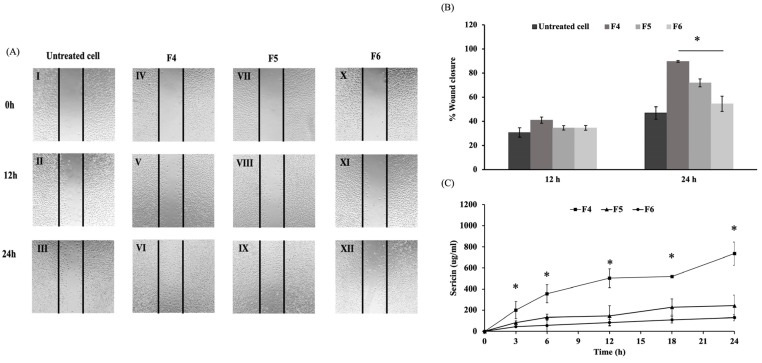
(**A**) Scratch assay of L929 cell line treated with F4, F5, and F6 supernatant at 12 and 24 h. I-III: Untreated cells, IV-VI: F4, VII-IX: F5, and X-XII: F6. Black lines mark the margins of the wound at 0 h in each photomicrograph. (**B**) Quantitative analysis of scratch closure after 12 and 24 h. (**C**) Sericin release from the F4, F5, and F6 formulations immersed in PBS at different times, with absorbance measured at 420 nm (* *p* < 0.05 compared to F4).

Fibroblasts in healthy skin are essential for connective tissue regeneration and the remodeling of tissues. Sericin facilitates the synthesis of type I collagen, which plays a critical role in collagen biosynthesis and is fundamental to the wound-healing process [27]. In this study, the scratch assay was applied to evaluate the effects of film-forming gel on fibroblast cell migration. The progression of wound closure was monitored at 12 and 24 h post-scratching and expressed as the percentage of wound healing over time (Figure 5A,B). The F4 formulation exhibited the most effective in cell migration, indicating enhanced wound healing compared to untreated cells and revealed the highest percentage of wound closure after 24 h at 89%, demonstrating a significant increase compared to F5 and F6 (*p* < 0.05). Previous studies found that sericin effectively stimulated fibroblast proliferation and migration by increasing cell adhesion and promoting mitogenic effects on mammalian cells [46,47]. This enhancement positively contributed to the wound-healing process, facilitating improved tissue regeneration and repair. Sericin release (Figure 5C) from the F4 formulation was significantly higher than from the F5 and F6 formulations (*p* < 0.05). A gradual release was sustained over 24 h, as one characteristic desired for wound treatment applications [48]. These findings suggested that the higher sericin content in F4 was associated with enhancing wound closure. Therefore, F4 showed promise as a biomaterial that effectively promoted wound healing.

#### 3.2.2. Effects on Inflammatory Response

**Figure 6:**
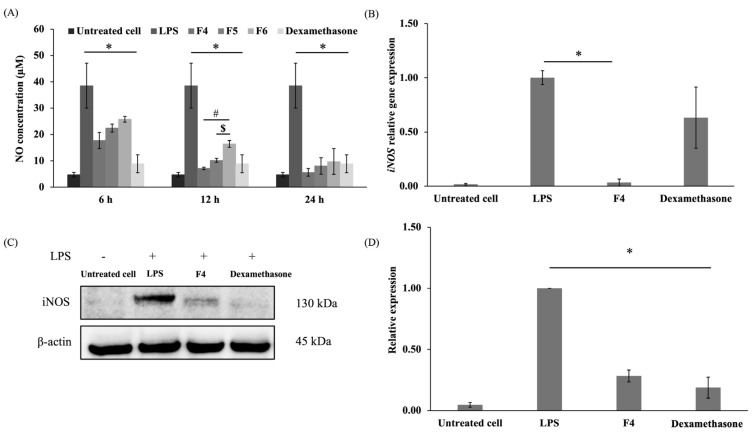
Anti-inflammatory effects of film-forming gel formulations on LPS-induced RAW 264.7 cells. (**A**) NO production, (**B**) *iNOS* gene expression normalized to *GAPDH*, (**C**) Expression of iNOS determined by Western blot analysis, (**D**) iNOS protein expression normalized to β-actin (* *p* < 0.05; film-forming gel formulations compared to LPS-stimulated RAW 264.7 cells, ^#^ *p* < 0.05; F4 compared to F5 and F6, ^$^ *p* < 0.05; F5 compared to F6).

Nitric oxide (NO) is a signaling molecule that plays a crucial role in regulating a wide range of cellular processes in the human body under normal physiological conditions and in pathological states. These processes include vasodilation, neurotransmission, inflammation, apoptosis, and tumorigenesis [49]. Elevated levels of nitric oxide trigger immune responses and drive the inflammatory cascade during inflammatory conditions. Activated macrophages stimulate the expression of inducible nitric oxide synthase (iNOS), which subsequently leads to the production of NO [50]. The inhibitory effects of the formulations on NO synthesis were evaluated in LPS-stimulated RAW 264.7 cells, with the F4, F5, and F6 formulations identified as promising anti-inflammatory agents. As shown in Figure 6A, NO production was almost undetectable in the unstimulated cells, whereas the LPS significantly induced NO production in RAW 264.7 cells compared to the untreated cells (*p* < 0.05). Dexamethasone, a commonly used drug for the treatment of inflammatory conditions, was used as a positive control in the experiment [51]. Dexamethasone significantly reduced the NO level compared to the LPS-treated cells (*p* < 0.05). The F4, F5, and F6 formulations significantly reduced NO in the LPS-induced cells at 6, 12, and 24 h compared to LPS-treated cells (*p* < 0.05). F4 significantly reduced NO production compared to the F5 and F6 formulations at 12 h (*p* < 0.05), while F5 demonstrated a significant decrease in NO compared to F6 at 12 h (*p* < 0.05). Therefore, the F4 formulation at 12 h, which strongly suppressed LPS-induced NO production in RAW 264.7 cells, was chosen for subsequent experiments in gene and protein expression.

The mRNA expression of *iNOS* was significantly upregulated in the LPS-induced macrophage cells (*p* < 0.05) (Figure 6B). A previous study found that dexamethasone reduced the mRNA expression of *iNOS* by inhibiting NF-κB and AP-1, transcription factors that regulate *iNOS* expression in the inflammatory process [51]. However, dexamethasone does not directly bind to *iNOS.* The F4 formulation significantly downregulated *iNOS* expression levels compared to the LPS-stimulated cells (*p* < 0.05), demonstrating that sericin in the formulation possessed a potent anti-inflammation capacity.

iNOS is a crucial enzyme in the inflammatory process that regulates multiple key mechanisms associated with intracellular signaling in different cells or species [52,53]. In macrophages, iNOS generates excessive NO in response to inflammation induced by LPS [50]. As shown in Figure 6C,D, the level of iNOS protein was upregulated in LPS-stimulated cells. By contrast, the F4 formulation and dexamethasone significantly suppressed iNOS protein expression (*p* < 0.05). These results confirmed the biological activity of sericin, which plays a significant role in modulating the inflammatory response by reducing the expression of iNOS.

**Figure 7:**
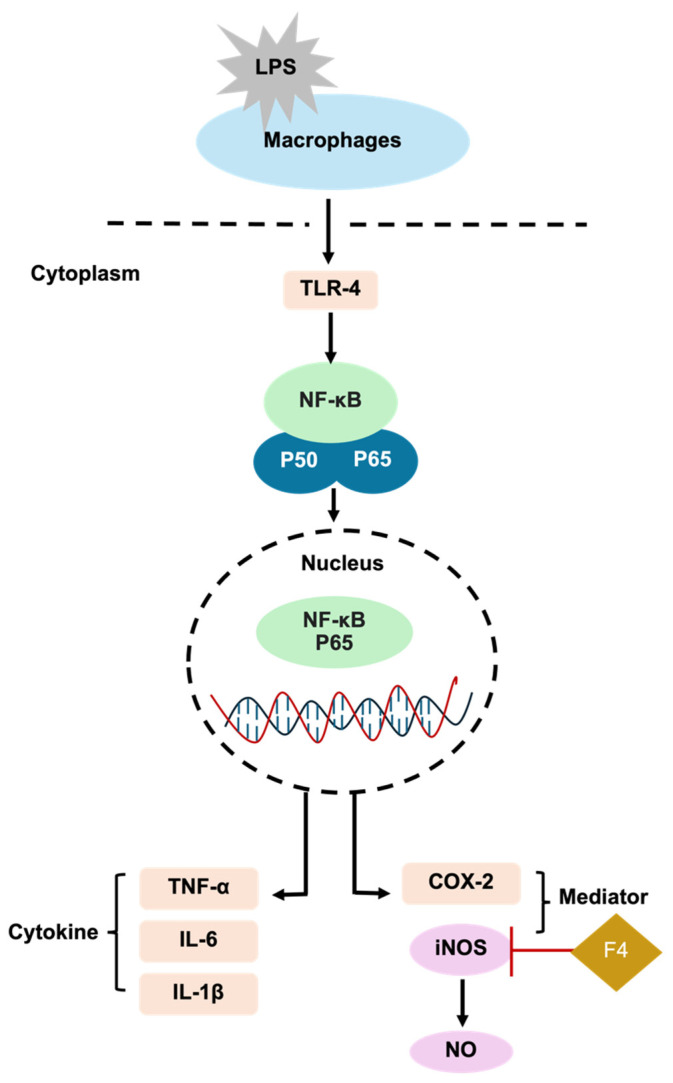
Proposed anti-inflammatory mechanism of the F4 formulation in suppressing iNOS in LPS-induced RAW 264.7 macrophage cells.

Inflammation is a biological defense mechanism that may occur in response to wound infection and tissue damage, leading to the generation of new tissue to repair damage [46]. Nuclear factor kappa B (NF-κB) controls the duration of inflammation by regulating cytokines and iNOS [54]. The suppression of NF-κB activity decreases *iNOS* transcription and subsequently reduces the excessive production of NO in macrophages [55]. Previous studies demonstrated that sericin inhibited iNOS expression [13,56]. Sericin reduced the production of pro-inflammatory cytokines including TNF-α, IL-1β, and IL-6 which are critical upstream regulators of iNOS [57]. The F4 formulation containing 1% sericin showed significant anti-inflammatory effects by suppressing the iNOS protein, thereby reducing *iNOS* gene expression and NO production (Figure 7).

## 4. Conclusions

This study evaluated sericin-based film-forming gel formulations for wound dressing applications, focusing on their physical and biological properties. The F4 formulation containing 1% sericin demonstrated superior drying time, spreadability, stability, and swelling ratio. The F4 formulation also exhibited excellent compatibility with skin fibroblast cells, maintaining pH levels within the acceptable range of 3.54 ± 0.52. Biological evaluations revealed that F4 significantly promoted cell proliferation and migration and exhibited anti-inflammatory effects by inhibiting iNOS expression and nitric oxide production. These findings highlighted F4 as a promising candidate for wound-healing applications, showing effective anti-inflammatory and regenerative properties.

## Figures and Tables

**Table 1:** Contents of sericin-based film-forming gel.

Formulations	Component Concentrations (%*w*/*v*)					
	10% PVA	0.25% Sericin	0.5% Sericin	1% Sericin	95% Ethanol	Glycerol
F1	6.5	-	-	0.2	7.9	6.3
F2	6.5	-	0.1	-	7.9	6.3
F3	6.5	0.05	-	-	7.9	6.3
F4	6.5	-	-	0.15	11.85	6.3
F5	6.5	-	0.075	-	11.85	6.3
F6	6.5	0.0375	-	-	11.85	6.3

**Table 2:** Primers used in this study.

Primer	Sequence (5′-3′)	Reference
*iNOS*	Forward: CACCACCCTCCTTGTTCAAC Reverse: CAATCCACAACTCGCTCCAA	[21]
*GAPDH*	Forward: CATGGCCTTCCGTGTTCCTA Reverse: CCTGCTTCACCACCTTCTTGAT	

**Table 3:** Characteristics of different sericin-based film-forming gel formulations.

Formulation	Integrity on Skin	Drying Time (min)		pH	Spreadability (g cm^2^/s)
		On Skin	On Glass Slide		
F1	a	4.29 ± 0.45	9.06 ± 0.22	5.22 ± 0.03	1.48 ± 0.13
F2	a	4.54 ± 0.27	8.28 ± 0.20	5.25 ± 0.02	1.59 ± 0.04
F3	a	4.43 ± 0.30	9.31 ± 0.05	5.23 ± 0.01	1.98 ± 0.10
F4	a	3.54 ± 0.52	6.54 ± 0.18	5.30 ± 0.01	1.54 ± 0.03
F5	a	4.18 ± 0.05	7.05 ± 0.15	5.37 ± 0.02	2.39 ± 0.06
F6	a	4.18 ± 0.21	6.35 ± 0.26	5.36 ± 0.02	2.31 ± 0.04

Note: a = complete film with no cracking or flaking.

## Data Availability

The raw data are available from the corresponding author by request.
